# Using Metabolomics to Differentiate Player Positions in Elite Male Basketball Games: A Pilot Study

**DOI:** 10.3389/fmolb.2021.639786

**Published:** 2021-05-13

**Authors:** Kayvan Khoramipour, Abbas Ali Gaeini, Elham Shirzad, Kambiz Gilany, Karim Chamari, Øyvind Sandbakk

**Affiliations:** ^1^ Department of Physiology and Pharmacology, Afzalipour Medical Faculty, and Physiology Research Center, Kerman University of Medical Sciences, Kerman, Iran; ^2^ Department of exercise physiology, University of Tehran, Tehran, Iran; ^3^ Department of health and sports medicine, University of Tehran, Tehran, Iran; ^4^ Reproductive Biotechnology Research Center, Academic Center for Education, Culture and Research, Avicenna Research Institute, Tehran, Iran; ^5^ ASPETAR, Qatar Orthopaedic and Sports Medicine Hospital, Doha, Qatar; ^6^ Centre for Elite Sports Research, Department of Neuromedicine and Movement Science, University of Science and Technology, Trondheim, Norway

**Keywords:** internal load, external load, team sport, metabolome, metabolites

## Abstract

**Purpose:** The current study compared metabolic profiles and movement patterns between different player positions and explored relationships between indicators of internal and external loads during elite male basketball games.

**Methods:** Five main players from 14 basketball teams (*n* = 70) were selected as subjects and defined as backcourt (positions 1–3) or frontcourt (positions 4–5) players. Video-based time motion analysis (VBTMA) was performed based on players’ individual maximal speeds. Movements were classified into high and low intensity running with and without ball, high and low intensity shuffling, static effort and jumps. Saliva samples were collected before and after 40-min basketball games with metabolomics data being analyzed by multivariate statistics. Independent *t*-tests were used to compare VBTMA.

**Results:** Frequency, duration, and distance of high and low intensity running and -shuffling were higher in backcourt players, whereas static effort duration and frequency as well as jump frequency were higher in frontcourt players (all *p* ≤ 0.05). The levels of taurine, succinic acid, citric acid, pyruvate, glycerol, acetoacetic acid, acetone, and hypoxanthine were higher in backcourt players, while lactate, alanine, 3-methylhistidine were higher and methionine was lower in frontcourt players (all *p* < 0.05). High intensity running with ball was significantly associated by acetylecholine, hopoxanthine, histidine, lactic acid and leucine in backcourt players (*p* < 0.05).

**Conclusion:** We demonstrate different metabolic profiles of backcourt and frontcourt players during elite male basketball games; while aerobic metabolic changes are more present in backcourt players, frontcourt players showed lager changes in anaerobic metabolic pathways due to more static movements.

## Introduction

Basketball is an intermittent team sport, characterized by alternating phases of low and high intensity work during four 10-min quarters. Players combine explosive and powerful accelerations, jumps, sprints, and specific movements such as rebounding, jump shooting, lay-ups, closing out, shot blocking, high-speed play, and fast breaks (Vázquez-Guerrero et al., 2019). Several factors can affect the movement demands in basketball, with player position being the most important (Pion et al., 2018) because each position is associated with its own duties and therefore requires specific movements. For example, backcourt players (i.e., point guards and shooting guards) run more than frontcourt players (i.e. centers) (Scanlan et al., 2011; Scanlan et al., 2012; Scanlan et al., 2014a). In contrast, point guards run more compared to forwards and centers (Hůlka et al., 2013). These differences reflect the nature of the positions. While backcourt players are generally smaller, quicker, and responsible for ball transitions, which require more movements, frontcourt players tend to be bigger, slower, and have more physical contact as they play in a smaller area (Kucsa and Mačura, 2015; Ibáñez et al., 2018). Such data is a result of external load monitoring studies using different methods of analysis among which video-based time motion analysis (VBTMA) is the most popular. However, these studies are based on predetermined speed thresholds that could result in over/under-estimate external load since player’s running speed vary depending on their playing levels, position, physical fitness, genetic background and other factors. Therefore, individualization of thresholds would make VBTMA a more accurate, valid approach (Abt et al., 2020).

The difference in movement patterns between different player positions indicates various contribution from aerobic and anaerobic metabolic pathways in each position during basketball games (Ferioli et al., 2018). In a previous study, the metabolic load of different positions (2 guards, 3 forwards, and 3 centers) in five games was compared, showing that guards have higher heart rates (HR) than forwards or centers (Vaquera et al., 2008). However, it is likely that HR underestimates the metabolic load in basketball because of its intermittent nature (Lambert and Borresen 2010), meaning that better methods to study the metabolic load are required. In this context, metabolomics can be a good alternative since it is a comprehensive, non-invasive approach, which can identify high numbers of metabolites simultaneously (Pohjanen et al., 2007). To date, previous studies have used metabolomics to distinguish the metabolic profile of athletes from different disciplines (Al-Khelaifi et al., 2018). However, the only study using metabolomics to investigate team sports is a comparison between the quarters of a game in elite male basketball players (Khoramipour et al., 2020).

The association between movement patterns determined by VBTMA and metabolic profiles assessed by metabolomics would provide a further understanding of the interaction between player movement and the metabolic consequences. Currently, only three studies have explored the correlation between indicators of internal and external loads in basketball games. Of these, Scanlan et al. (2014b) found moderate correlations between accelerometer data and session ratings of perceived exertion (sRPE), training impulse (an index coming from a combination of heart rate during training and rest) and summated-heart-rate-zones (adding up the time spent in each of five HR zones) in semiprofessional male basketball players. Furthermore, Svilar et al. (2018) reported that player load, acceleration and deceleration, jumps, and changes of direction were highly correlated to sRPE. More detailed internal load analyses (e.g., using metabolomics) could provide a better understanding of the associations between external and internal load in basketball.

Therefore, the current pilot study aimed to compare metabolic profiles and movement patterns between different player positions during elite male basketball games. In addition, we explored the relationships between metabolic profiles and movement patterns as indicators of internal and external loads. We hypothesize that backcourt players have higher movement frequency, duration, and distance and show larger changes in aerobic metabolites, while metabolic changes in frontcourt players tend to be more anaerobic due to the nature of their positions, which require less dynamic but more static, strength and power movement patterns.

## Materials and Methods

### Subjects

The data presented in this study is part of a larger investigation of male basketball games where one study is published previously **(** Khoramipour et al., 2020). Five main players across 14 teams (*N* = 70) who participated in the 2017–18 Iranian national basketball super league (e.g., top division) were randomly selected as subjects (age: 24.2 ± 5.3 years, height: 192.4 ± 12.3 cm, weight: 88.7 ± 6.4 kg, body fat percentage: 10.9 ± 1.3, BMI: 24.1 ± 0.8 kg/m^2^) and studied during seven games (two teams and a total of 10 players in each game). Having at least 3 years league experience was considered as inclusion criteria; however, international players were excluded. Subjects reported no metabolic disorder and were asked to refrain from taking supplements one month prior to the study.

### Ethics Statement

The Research Ethics Committee of the University of Tehran (IR.UT.SPORT.REC. 1398.007) approved this study. Prior to the data collection, all subjects provided written informed consent to voluntarily take part in the study. The subjects were informed that they could withdraw from the study at any point in time without providing a reason for doing so.

### Design

In order to study metabolic profiles and movement demands in different positions during elite male basketball games, we experimentally combined saliva metabolomics analyses to non-invasively examine internal load and VBTMA to study external load, with the players movements classified into eight categories based on athletes’ maximal shuffling and running speeds. Saliva samples were employed due to the combination of easy sampling procedure and informative, stable measurements as described elsewhere (Dame et al., 2015). Samples were collected before and after each game and analyzed using Hydrogen nuclear magnetic resonance (HNMR). In addition, we explored the associations between these measures of internal and external load using linear regression models.

### Methodology

Initially, the study procedure was explained to coaches and players and coaches were asked to decrease training load by 50% in the last three days prior to the beginning of the study. Thereafter, players’ body height, body weight, body fat percentages (using 7-site measurements using a caliper), as well as personal information were recorded. 3–5 days before the games, individual maximal running and shuffling tests were performed. The games were held based on the International Basketball Federation (FIBA) rules; each game consisted of four quarters (10 min each) with a 2-min rest interval between the first and second quarters (i.e., first half) and between the third and fourth quarters (i.e., second half). There were 15 min breaks between the halves. The games were held 2 weeks before the competitive season started to avoid interference from other games. All games started at 11:00 a.m. and players arrived at the stadium at 8:00 a.m. to consume a normal breakfast consisting of around 400 calories (60% carbohydrate, 30% protein, and 10% fat), which was calculated using food processor nutrition analysis software (PCN software; Cesnid, Spain). After breakfast, subjects brushed their teeth and were not allowed to eat to avoid physiological interference with saliva metabolites. The subjects drank 200 ml of water prior the games and the same amount in each half. On their arrival at the stadium, subjects were asked to sit down for 30 min to recover from possible metabolic turbulence resulting from daily activity. This was done to control for the main influencers on saliva metabolome (i.e., before training meal, saliva reaction with mouse components, hydration status, and daily physical activity).

Thereafter, they started their regular warm-up protocol consisting of running, dribbling, shots, and passes. All subjects played for 40 min and no substitutions were allowed. Saliva samples were collected from each player before and after the games. To collect saliva, the subjects spat in a 15 ml falcon until at least 3 ml saliva was collected. The samples were placed on liquid nitrogen and kept at -80°C until analysis. At the same time, the players external load was also assessed using VBTMA (*see* details below). Due to position changes during the game, positions 1, 2, and 3 were considered as backcourt players and 4 and 5 as frontcourt players. Accordingly, a total of 70 players were divided into 42 backcourt and 28 frontcourt players. This classification was based on the available data and our pilot study which showed similar movement patterns for positions 1, 2, and 3 as well as for positions 4 and 5.

### NMR Sample Preparation

After sampling, the saliva samples were placed at ambient temperature to be liquefied, and then centrifuged for 20 min (11,200 RCF, 4°c) to remove supernatant. Thereafter, 450 μl of the samples were dissolved in 150 μl of D2O, transferred to 5 mm high quality tubes, and put in the 500 MHz Bruker DRX HNMR (Bruker company, Madison, United States).

### NMR Data Acquisition

Spectroscopy was conducted using the Carr-Purcell-Meiboom-Gill (CPMG) method. CPMG reduces wide protein resonances and helps increase the resolution of low molecular weight metabolites. All spectra referred to methyl lactate (1.33 ppm). Other spectroscopy parameters were as follows: temperature: 298 K, scan number: 154, peak width: 8389.26 Hz, and pulse time: 2 s.

### Spectra Processing

The MestreNova (MasterLab company, Santiago De Compostela, Spain) software was used for spectra processing. In a first step, the water peak was deleted, followed by baseline correction, phase correction, and normalization. Then, all data were converted into numbers with each sample consisting of 408 numbers. The data were sorted in Microsoft Excel (Microsoft company, United States) and uploaded using the web-based software MetaboAnalyst (www.metaboanalyst.ca).

### Individual Run and Shuffle Speed Tests

To assess maximal running speed, a 30-yard speed test was performed using an indoor basketball court. To assess maximal shuffling speed, a 15-yard shuffle test was performed. This test included a 15-yard right shuffle and a 15-yard left shuffle to return to the starting point. Running and shuffling speeds were recorded with a statistic camera (Basler A602FC; Basler Vision Technologies, Germany), and maximal speed was calculated using Dartfish 10 Pro (Dartfish company, Fribourg, Switzerland).

### Video-Based Time Motion Analysis

The games were recorded with two fixed wide-angle color cameras (Basler A602FC; Basler Vision Technologies, Germany), mounted at a ∼15 m height at the middle of the half court and ∼9 m distance from the sideline. Players were recorded for entire games (including all stoppages), and mean frequency, duration (s), and distance (m) were calculated for each activity category (Table 1). Recordings were analyzed by Dartfish 10 Pro and normalized for the individual’s maximal speed (Table 1). Two movement classes were simply analyzed by watching the film: 1) static efforts and 2) jumps. Dartfish 10 Pro is a 3D software, which can resolve the perspective problem. To avoid inter-observer variability, a single experienced observer analyzed all games. Before the study, the observer analyzed two quarters of the same game one month apart, reporting high test-retest reliability (correlation = 0.85).

**TABLE 1:** The seven movement categories (based on each player’s maximal speed) are used for categorization in the current study.

Movement	Speed
Low intensity running without ball (LIRWO)	≤70% player’s maximal speed
Low intensity running with ball (LIRW)	≤70% player’s maximal speed
High intensity running without ball (HIRWO)	>70% player’s maximal speed
High intensity running with ball (HIRW)	>70% player’s maximal speed
Low intensity shuffling (LISH)	≤70% player’s maximal shuffle speed
High intensity shuffling (HISH)	>70% player’s maximal shuffle speed
Static effort (SE)	-
Jump (JU)	-

### Statistical Analysis

Descriptive statistics (mean ± SD) were calculated for each movement variables (VBTMA). Thereafter, the Kolmogorov–Smirnov test supported the use of parametric analyses, in which the independent *t*-test was used to study the difference between positions in movement patterns. PCA (Principal component analysis) and PLSDA (partial least squares discriminant analysis) were used to compare the metabolome between positions and *t*-tests to identify significantly different bins (Chong et al., 2018). Thereafter, the bins were entered in the human metabolome database (http://www.hmdb.ca) to identify different bins associated metabolites. A linear regression model was employed to study the association between indicators of internal and external load, by setting each movement class (Table 1) as a dependent variable and the various metabolites as independent variables using the enter method. The regressions were done within each position separately, and we initially included all metabolites with significant changes in each position as independent variables. The alpha level was set to *p* ≤ 0.05.

## Results

### Time Motion Analysis

Movement frequency, duration, and distance are presented in Table 2. For frequency, duration, and distance, high intensity running with ball, high intensity running without ball, low intensity running with ball, low intensity running without ball, high intensity shuffling, and low intensity shuffling were higher in backcourt players, whereas static movement frequency and duration as well as jump frequency were higher among frontcourt players (*p* ≤ 0.05 for all).

**TABLE 2:** Frequency (#), duration (s), and distance (m) of the seven movement categories for the backcourt (*N* = 42) and frontcourt (*N* = 28) players during elite male basketball games analyzed by video-based time motion analysis (mean ± SD).

Motion Variable	Position	High intensity running with ball	High intensity running without ball	Low intensity running with ball	Low intensity running without ball	High intensity shuffle	Low intensity shuffle	Static efforts	Jump
Frequency	backcourt	43 ± 4^a^	92 ± 27^a^	86 ± 8^a^	348 ± 24^a^	66 ± 6^a^	77 ± 5^a^	69 ± 4^a^	41 ± 6
	frontcourt	31 ± 6	80 ± 9	34 ± 9	193 ± 13	22 ± 4	21 ± 4	190 ± 6	79 ± 5
Duration (s)	backcourt	101 ± 10^a^	301 ± 73^a^	320 ± 33^a^	1656 ± 63^a^	142 ± 14^a^	163 ± 16^a^	105 ± 9^a^	60 ± 6
	Frontcourt	72 ± 8	251 ± 53	134 ± 8	840 ± 52	71 ± 14	51 ± 13	323 ± 29	114 ± 16
Distance (m)	backcourt	715 ± 78^a^	2486 ± 235^a^	762 ± 45^a^	3981 ± 147^a^	289 ± 27^a^	271 ± 32^a^	-	-
	frontcourt	350 ± 56	1420 ± 105	346 ± 63	1863 ± 120	115 ± 19	85 ± 9	-	-

a Significant difference between positions.

### Metabolomics

**FIGURE 1:**
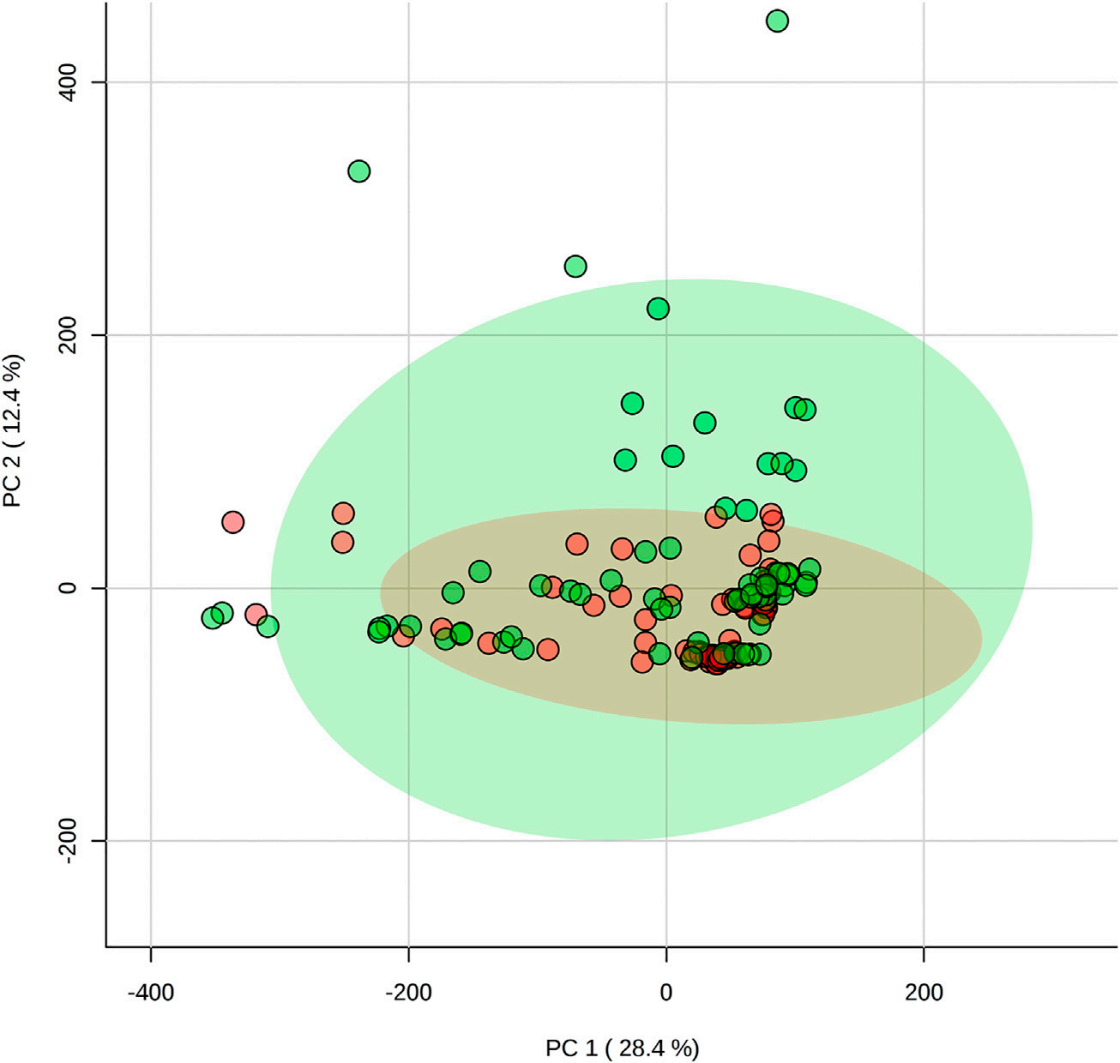
Comparison of pre-game metabolomes (metabolic profile) between backcourt and frontcourt players during elite male basketball games using principal component analysis. Red: backcourt, green: frontcourt.

**FIGURE 2:**
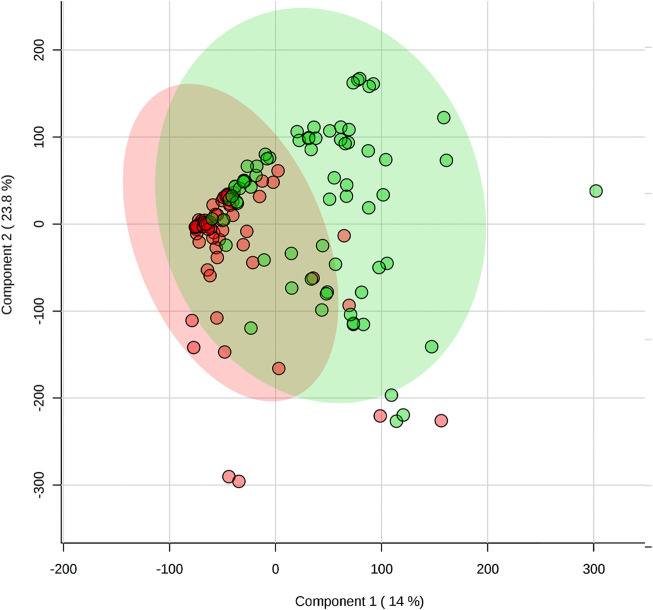
Comparison of pre-game metabolomes (metabolic profile) between backcourt and frontcourt players during elite male basketball games using partial least square discriminant analysis. Red: backcourt, green: frontcourt.

The saliva samples collected before the games were compared with PCA (Figure 1) and PLSDA (Figure 2) and showed no significant differences between positions.

**FIGURE 3:**
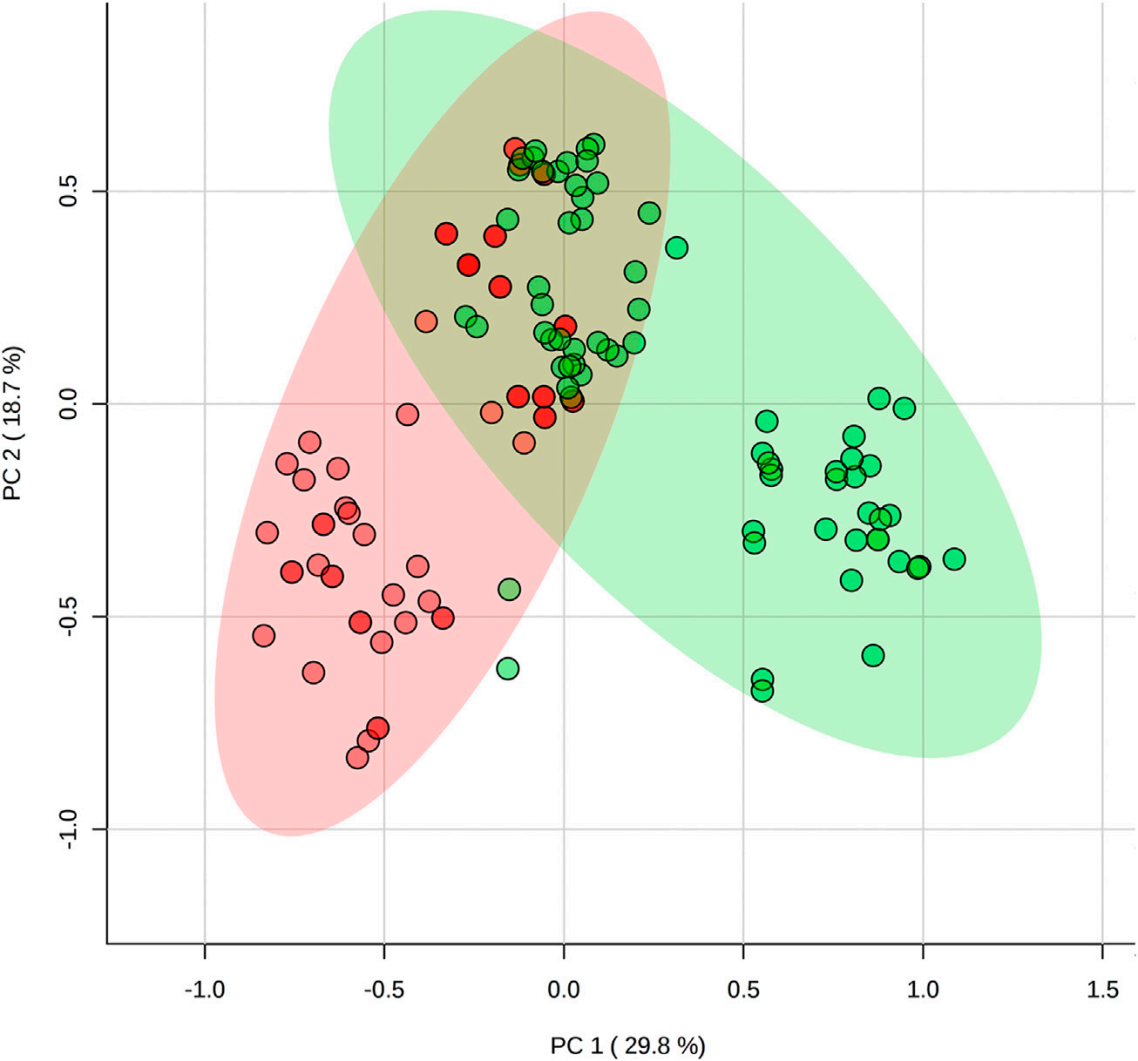
Comparison of post-game metabolomes (metabolic profile) between backcourt and frontcourt players during elite male basketball games using principal component analysis. Red: backcourt, green: frontcourt.

**FIGURE 4:**
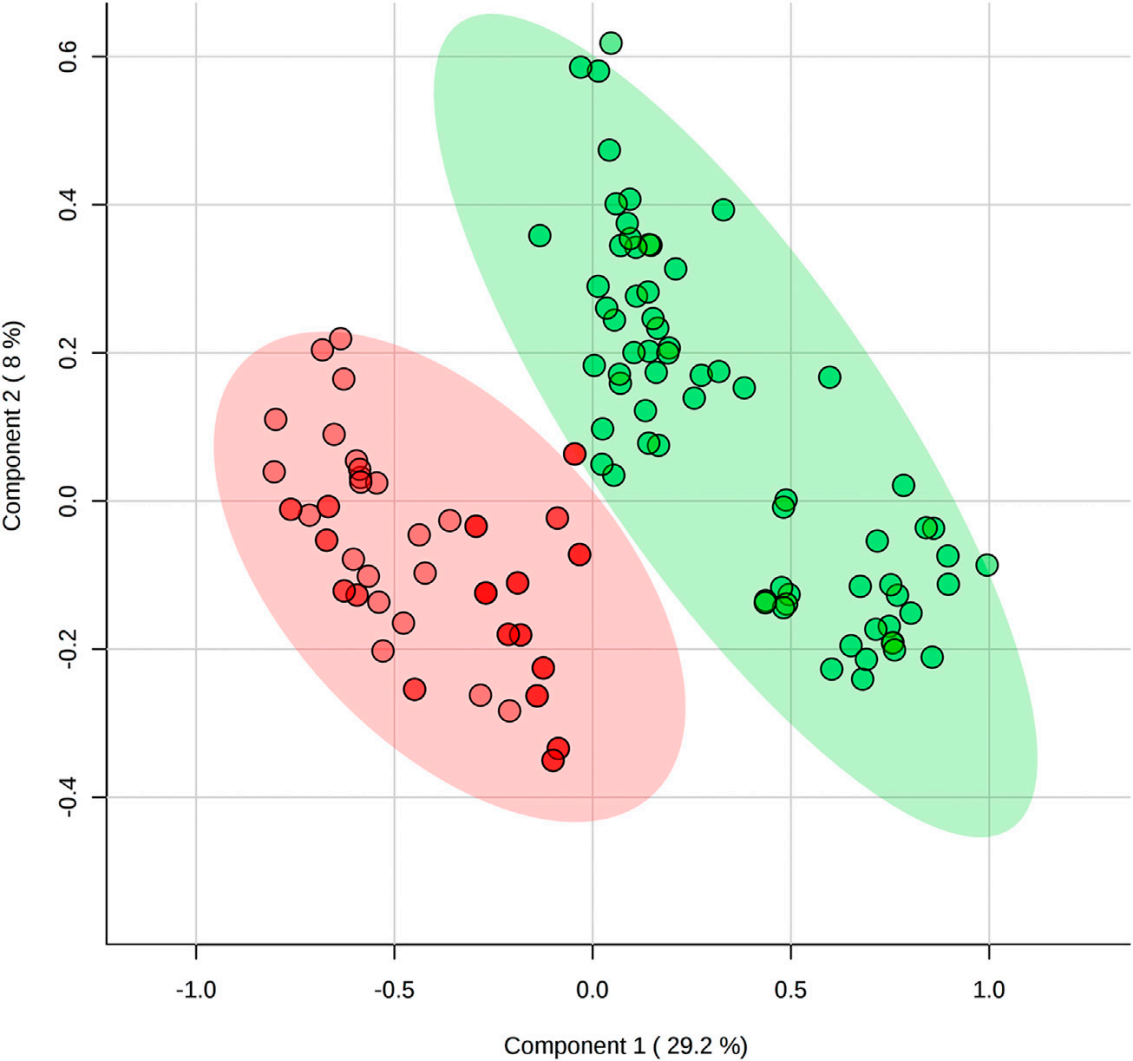
Comparison of post-game metabolomes (metabolic profile) between backcourt and frontcourt players during elite male basketball games using partial least square discriminant analysis. Red: backcourt, green: frontcourt.

Post-game comparisons in metabolomes showed several differences between positions as illustrated in the PCA (Figure 3) and PLSDA plots (Figure 4). The levels of taurine, succinic acid, citric acid, pyruvate, glycerol, acetoacetic acid, acetone, and hypoxanthine were higher in backcourt players, while lactate, alanine, 3-methylhistidine were higher and methionine was lower in frontcourt players (all *p* < 0.05).

The metabolites with significant pre-to post-game changes are listed in Table 3.

**TABLE 3:** Metabolites showing significant differences between backcourt and frontcourt players in the post-game samples during elite male basketball.

Metabolites	T-value	*p*-value	−Log 10 (p)	FDR
Taurine	−4.24	0.00^a^	3.99	0.00
Succinic acid	−4.19	0.00^a^	3.92	0.00
Citric acid	−4.15	0.00^a^	3.87	0.00
Pyruvate	−4.15	0.00^a^	3.86	0.00
Glycerol	4.10	0.00^a^	3.80	0.00
Acetoacetic acid	−4.08	0.00^a^	3.78	0.00
Acetone	−4.05	0.00^a^	3.73	0.00
Hypoxanthine	−4.03	0.00^a^	3.70	0.00
Lactate	−4.01	0.00^b^	3.68	0.00
Alanine	−4.00	0.00^b^	3.67	0.00
3-methylhistidine	3.97	0.00^b^	3.63	0.00
Methionine	−3.96	0.00^&^	3.60	0.00

a Significantly higher levels in backcourts.

b significantly higher levels in frontcourts, $ significantly lower levels in frontcourts. FDR, false discovery rate.

### Correlation Between Internal and External Load

The only significant prediction of VBTMA from metabolomics indicators was found for high intensity running with ball (HIRW) (*p* = 0.05, *R* ^2^ = 0.866) (Tables 4 and 5). The final regression formula is as follow:

**TABLE 4:** Prediction of the various movements classes from metabolites using regression analysis in backcourt players.

Movement frequency	F-value	*p*-value	*R* ^2^
High intensity running with ball	2.54	0.05^a^	0.86
High intensity running without ball	2.41	0.06	0.86
Low intensity running with ball	1.53	0.23	0.79
Low intensity running without ball	0.47	0.94	0.54
High intensity shuffle	0.80	0.69	0.67
Low intensity shuffle	0.81	0.68	0.67
Static efforts	2.30	0.07	0.85
Jump	0.84	0.65	0.68

a All metabolites with significant changes in each position were initially included as independent variables.

**TABLE 5:** Prediction of the various movements classes from metabolites using regression analysis in frontcourt players.

Movement frequency	F-value	*p*-value	*R* ^2^
High intensity running with ball	1.55	0.36	0.90
High intensity running without ball	0.47	0.88	0.74
Low intensity running with ball	1.14	0.50	0.87
Low intensity running without ball	0.75	0.68	0.81
High intensity shuffle	1.12	0.51	0.87
Low intensity shuffle	2.43	0.20	0.93
Static efforts	0.38	0.93	0.70
Jump	1.23	0.46	0.88

All metabolites with significant changes in each position were initially included as independent variables.

HIRW = 111.895 + 6.880 acetylecholine − 12.414 hopoxanthine − 7.430 histidine +4.731 lactic acid − 6.289 leucine.

## Discussion

The present study shows that movement frequency, duration, and distance of high and low intensity running and -shuffling were higher in backcourt players, whereas static effort frequency and duration as well as jump frequency were higher in frontcourt players. Accordingly, specific indicators of aerobic metabolism such as taurine, succinic acid, citric acid, pyruvate, glycerol, acetoacetic acid, acetone, and hypoxanthine were higher in backcourt players, while indicators of anaerobic metabolism such as lactic acid, alanine, and 3-methylhistidine were higher and methionine was lower in frontcourt players. Furthermore, a regression analysis shows that acetylcholine, hypoxanthine, histidine, lactic acid and Leucine could significantly predict HIRW.

Logically following time motion analysis showing that backcourt players spend more time doing dynamic movements during the game, the coinciding metabolomic analysis indicates a greater reliance of aerobic metabolism compared to frontcourts. More specifically, we find higher pyruvate concentration and lower lactic acid in backcourt players which indicate that most of the produced pyruvate entered the Krebs cycle and was utilized in the aerobic metabolism (Mougios 2019; Schenk et al., 2021). A higher Krebs cycle activity is also supported by higher succinic acid and citric acid (i.e., Krebs cycle mediators) (Blackburn et al., 2020). A previous study showed that VO_2max_ was higher in backcourt than in frontcourt players (Abdelkrim et al., 2007), which is congruent with our data. Another study where %HR_peak_ was used to compare the metabolic load between different positions showed that guards’ (considered as backcourt players in our approach) mean HR corresponded to 88% of HR_peak_ (Hůlka et al., 2013), showing high reliance on aerobic pathways. However, HR cannot be considered a valid measure of basketball load monitoring since it may underestimate physiological load. Here, we instead assessed Krebs cycle mediators, which are aerobic metabolism indexes, directly (Mougios 2019) and therefore a more valid approach. Furthermore, higher levels of hypoxanthine, the end product of the purine cycle, further support a higher aerobic energy turnover and oxidative stress (Zhao et al., 2020) in backcourt players; and increased energy demand further led to increased purine cycle activity and subsequently higher hypoxanthine (Mougios 2019). In addition, taurine, the most abundant amino acid-like component in muscle and other organs, was higher in backcourt players (Huffman et al., 2014). We also found higher amounts of ketone bodies (e.g., acetoacetic acid and acetone) and glycerol as indexes of higher fat oxidation in backcourt players. As a practical consequence of the above findings of higher aerobic pathway activity and fat oxidation with more dynamic movements, a greater requirement for endurance training in backcourt players could be expected.

There are several explanations for the larger oxidative stress found in backcourt players. First, backcourt players perform more high and low intensity shuffling, which is considered to be the most intense movements in basketball (Abdelkrim et al., 2007) due to the consecutive changes of direction, acceleration, and deceleration (Abdelkrim et al., 2007; Scanlan et al., 2012). In this connection Schelling and Torres (2016) reported that guards (i.e., backcourts) show higher acceleration load mainly because of their lower body mass which allow them to accelerate rapidly. On the basis of the same reasoning, Reina et al. (2020) concluded that centers experienced lower overall load during the game. In contrast to our findings, Ferioli et al. (2020) reported a higher rate of high intensity movements as well as more time spent doing high intensity movements in centers (e.g., frontcourt players) than guards. Two main reasons could explain this controversy; they did not subdivide the various high intensity movements and included static efforts and jumps in this category. Here, our results show that the amount of static efforts and jumps are higher in centers. In addition, Ferioli et al. (2020) used qualitative methods for time motion analysis, in which intra-variability could be a source of error, in contrast to our more accurate quantitative analysis method.

Second, backcourt players’ roles are ball carrying and dribbling, since they are more skilled in ball handling (Scanlan et al., 2012) and in defending smaller players who perform more agile movements and changes of direction (Abdelkrim et al., 2007). This is supported by Ferioli et al. (2020) who showed that guards are the players with the highest ball processioning and when external load monitoring is conducted with ball possession, all movement categories are higher in guards. Backcourts are also the first players who start both offense and defense actions, resulting in more activity (Abdelkrim et al., 2007). In addition, previous studies using different approaches, such as sRPE, showed higher metabolic loads in smaller players (i.e. backcourt players) and especially point guards.

Third, backcourt players’ playing zone is out of the 3-point shot, which is less crowded and has more movement space, meaning that players can move more easily and thereby increase their activity. García et al. (2020) reported more covered distance in guards than centers who need to stay in close distance to the basket for rebounding. Playing in this small, crowed zone requires frontcourt players to have much body contact during rebound and positioning, which is evident by more static efforts (Abdelkrim et al., 2007; García et al., 2020) and leads to higher muscle damage clearly indicated by higher 3 mtyle histidine in frontcourt players. Accordingly, such intense physical activity is dependent on anaerobic rather than aerobic metabolism. This may also explain the higher alanine concentration in frontcourt players as alanine is the precursors of carnosine production, the main intracellular buffer.

In the current study, indicators of anaerobic metabolism such as lactic acid, alanine, and 3-methylhistidine were higher and methionine was lower in frontcourt players, indicating greater reliance of anaerobic metabolism. Previous studies have only measured lactate, in which higher lactate concentrations have been found in guards than forwards or centers (Rodriguez-Alonso et al., 2003; Abdelkrim et al., 2007). In contrast, Scanlan et al. (2011) did not observe such differences between positions, in which tactical approaches, player level, and playing style may explain the differences between these studies.

The only significant prediction of VBTMA data from metabolomics indicators found using regression analysis was for HIRW in backcourt players. This makes sense since HIRW is a demanding movement in basketball, which lead to the largest change in the metabolites (e.g., acetylcholine, hopoxanthine, histidine, lactic acid and leucine). Likely, the higher frequency in backcourt players lead to the significant correlation between HIRW and selected metabolites in these players. Although Fox et al. (2020) showed that player load (representing a measure of the accumulated load) was correlated with sRPE and session heart rate zone (sHRZ) in training sessions and games, our study was the first to correlate metabolomics as a measurement of the internal metabolic load, which provide more detailed insights than previous studies.

We had three main limitations in this study: First, we were not able to include the same diet for several days preceding the games. Since diet could affect the players’ metabolomes, our result should therefore be taken with some caution. Second, using the 500 MHz Bruker DRX HNMR, we may have missed some metabolic changes due to low power of the device. Finally, we explained the physiological basis for our findings, but the reader should be aware that subject properties (i.e. demographic and genetic), athletic experience, component of the team and game strategy could affect both internal and external loads, illustrating that our results should be interpreted with this in mind.

## Conclusion

Based on novel metabolomics analyses, we demonstrate clearly different metabolic profiles of backcourt and frontcourt players during elite male basketball games; while backcourt players move more and show more aerobic metabolite changes, frontcourt players represent more anaerobic changes due to more static movement patterns. However, the relationships between these indicators of internal and external load were modest and the only significant prediction of movement category found was HIRW based on acetylecholine, hopoxanthine, histidine, lactic acid and Leucine in backcourt players.

## Data Availability

The datasets presented in this study can be found in online repositories. The names of the repository/repositories and accession number(s) can be found in the article/Supplementary Material. All data associated with this study can be found here: https://sport.ut.ac.ir/.
